# Supplementary material for: Incidence and risk factors of systemic lupus erythematosus in patients with primary immune thrombocytopenia: a systematic review and meta-analysis
Source: PeerJ. 2024 Apr 22;12:e17152. doi: 10.7717/peerj.17152 (PMC11044880; doi:10.7717/peerj.17152)
Supplement: Supplemental Information 2 [file peerj-12-17152-s002.docx]

**Table S1** Literature search strategy

**1. Pubmed**

| Search number | Query | Results |
| --- | --- | --- |
| #1 | Purpura, Thrombocytopenic, Idiopathic[MeSH Terms] | 7726 |
| #2 | "Idiopathic Thrombocytopenic Purpura"[Title/Abstract] OR "Idiopathic Thrombocytopenic Purpuras"[Title/Abstract] OR "Immune Thrombocytopenic Purpura"[Title/Abstract] OR "Immune Thrombocytopenic Purpuras"[Title/Abstract] OR "Thrombocytopenic Purpura, Immune"[Title/Abstract] OR "Thrombocytopenic Purpuras, Immune"[Title/Abstract] OR "Immune Thrombocytopenia"[Title/Abstract] OR "Immune Thrombocytopenias"[Title/Abstract] OR "Werlhof Disease"[Title/Abstract] OR "Werlhof's Disease"[Title/Abstract] OR "Werlhofs Disease"[Title/Abstract] OR "Autoimmune Thrombocytopenia"[Title/Abstract] OR "Autoimmune Thrombocytopenias"[Title/Abstract] OR "Autoimmune Thrombocytopenic Purpura"[Title/Abstract] OR "Autoimmune Thrombocytopenic Purpuras"[Title/Abstract] | 12724 |
| #3 | Lupus Erythematosus, Systemic[MeSH Terms] | 67457 |
| #4 | "Lupus Erythematosus Disseminatus"[Title/Abstract] OR "Libman-Sacks Disease"[Title/Abstract] OR "Libman Sacks Disease"[Title/Abstract] OR "dermatovisceritism, malignant"[Title/Abstract] OR "disseminated lupus"[Title/Abstract] OR "disseminated lupus erythematodes"[Title/Abstract] OR "disseminated lupus erythematosus"[Title/Abstract] OR "erythematodes visceralis"[Title/Abstract] OR "lupovisceritis"[Title/Abstract] OR "lupus erythematodes disseminates"[Title/Abstract] OR "lupus erythematosus disseminates"[Title/Abstract] OR "lupus erythematosus visceralis"[Title/Abstract] OR "lupus erythematosus, systemic"[Title/Abstract] OR "osler libman sacks disease"[Title/Abstract] OR "systemic lupus erythematodes"[Title/Abstract] OR "systemic lupus erythematous "[Title/Abstract] | 3664 |
| #5 | (#1 OR #2) AND (#3 OR #4) | 386 |

**2. Cochrane**

| Search number | Query | Results |
| --- | --- | --- |
| #1 | MeSH descriptor: [Purpura, Thrombocytopenic, Idiopathic] explode all trees | 383 |
| #2 | ('autoimmune thrombocytopaenia' OR 'immune thrombocytopenia' OR 'thrombocytopaenia, autoimmune' OR 'thrombocytopenia, autoimmune' OR 'Evans syndrome' OR 'autoimmune hemolytic anemia and autoimmune thrombocytopenia' OR 'evan syndrome' OR 'Fisher-Evans syndrome' OR 'chronic idiopathic thrombocytopaenia' OR 'chronic idiopathic thrombocytopenia' OR 'idiopathic thrombocytopaenia' OR 'idiopathic thrombocytopenia' OR 'immune thrombocytopenic purpura' OR 'ITP' OR 'morbus werlhof' OR 'Werlhof disease'):ab,ti,kw | 1732 |
| #3 | MeSH descriptor: [Lupus Erythematosus, Systemic] explode all trees | 1448 |
| #4 | ('Lupus Erythematosus Disseminatus' OR 'Libman-Sacks Disease' OR 'Libman Sacks Disease' OR 'dermatovisceritism, malignant' OR 'disseminated lupus' OR 'disseminated lupus erythematodes' OR 'disseminated lupus erythematosus' OR 'disseminated lupus erythematosus' OR 'erythematodes visceralis' OR 'lupovisceritis' OR 'lupus erythematodes disseminates' OR 'lupus erythematosus disseminates' OR 'lupus erythematosus visceralis' OR 'lupus erythematosus, systemic' OR 'osler libman sacks disease' OR 'systemic lupus erythematodes' OR 'systemic lupus erythematous' OR 'systemic lupus erythematosus' OR 'Disease, Libman-Sacks '):ab,ti,kw | 2929 |
| #5 | (#1 OR #2) AND (#3 OR #4) | 30 |

**3. Embase**

| Search number | Query | Results |
| --- | --- | --- |
| #1 | 'lupus erythematosus disseminatus':ti,ab,kw OR 'libman-sacks disease':ti,ab,kw OR 'libman sacks disease':ti,ab,kw OR 'dermatovisceritism, malignant':ti,ab,kw OR 'disseminated lupus':ti,ab,kw OR 'disseminated lupus erythematodes':ti,ab,kw OR 'disseminated lupus erythematosus':ti,ab,kw OR 'erythematodes visceralis':ti,ab,kw OR 'lupovisceritis':ti,ab,kw OR 'lupus erythematodes disseminates':ti,ab,kw OR 'lupus erythematosus disseminates':ti,ab,kw OR 'lupus erythematosus visceralis':ti,ab,kw OR 'lupus erythematosus, systemic':ti,ab,kw OR 'osler libman sacks disease':ti,ab,kw OR 'systemic lupus erythematodes':ti,ab,kw OR 'systemic lupus erythematous':ti,ab,kw | 4508 |
| #2 | 'lupus erythematosus, systemic'/exp | 118631 |
| #3 | 'autoimmune thrombocytopenia'/exp | 27955 |
| #4 | 'autoimmune thrombocytopaenia':ab,ti,kw OR 'immune thrombocytopenia':ab,ti,kw OR 'thrombocytopaenia, autoimmune':ab,ti,kw OR 'thrombocytopenia, autoimmune':ab,ti,kw OR 'evans syndrome':ab,ti,kw OR 'autoimmune hemolytic anemia and autoimmune thrombocytopenia':ab,ti,kw OR 'evan syndrome':ab,ti,kw OR 'fisher-evans syndrome':ab,ti,kw OR 'chronic idiopathic thrombocytopaenia':ab,ti,kw OR 'chronic idiopathic thrombocytopenia':ab,ti,kw OR 'idiopathic thrombocytopaenia':ab,ti,kw OR 'idiopathic thrombocytopenia':ab,ti,kw OR 'immune thrombocytopenic purpura':ab,ti,kw OR 'itp':ab,ti,kw OR 'morbus werlhof':ab,ti,kw OR 'werlhof disease':ab,ti,kw | 19512 |
| #5 | (#1 OR #2) AND (#3 OR #4) | 1956 |

**4. Web of science**

| Search number | Query | Results |
| --- | --- | --- |
| #1 | ALL=("Idiopathic Thrombocytopenic Purpura" OR "Idiopathic Thrombocytopenic Purpuras" OR "Immune Thrombocytopenic Purpura" OR "Immune Thrombocytopenic Purpuras" OR "Thrombocytopenic Purpura, Immune" OR "Thrombocytopenic Purpuras, Immune" OR "Immune Thrombocytopenia" OR "Immune Thrombocytopenias" OR "Werlhof Disease" OR "Werlhof's Disease" OR "Werlhofs Disease" OR "Autoimmune Thrombocytopenia" OR "Autoimmune Thrombocytopenias" OR "Autoimmune Thrombocytopenic Purpura" OR "Autoimmune Thrombocytopenic Purpuras" OR "autoimmune thrombocytopaenia" OR "immune thrombocytopenia" OR "thrombocytopaenia, autoimmune" OR "thrombocytopenia, autoimmune" OR "Evans syndrome" OR "autoimmune hemolytic anemia and autoimmune thrombocytopenia" OR "evan syndrome" OR "Fisher-Evans syndrome" OR "chronic idiopathic thrombocytopaenia" OR "chronic idiopathic thrombocytopenia" OR "idiopathic thrombocytopaenia" OR "idiopathic thrombocytopenia" OR "immune thrombocytopenic purpura" OR "ITP" OR "morbus werlhof" OR "Werlhof disease" OR "Purpura, Thrombocytopenic, Idiopathic" OR "autoimmune thrombocytopenia") | 22875 |
| #2 | ALL=("Lupus Erythematosus Disseminatus" OR "Libman-Sacks Disease" OR "Libman Sacks Disease" OR "dermatovisceritism, malignant" OR "disseminated lupus" OR "disseminated lupus erythematodes" OR "disseminated lupus erythematosus" OR "disseminated lupus erythematosus" OR "erythematodes visceralis" OR "lupovisceritis" OR "lupus erythematodes disseminates" OR "lupus erythematosus disseminates" OR "lupus erythematosus visceralis" OR "lupus erythematosus, systemic" OR "osler libman sacks disease" OR "systemic lupus erythematodes" OR "systemic lupus erythematous" OR "systemic lupus erythematosus" OR "Disease, Libman-Sacks" OR "Systemic Lupus Erythematosus" OR "Lupus Erythematosus, Systemic") | 84496 |
| #3 | #2 AND #1 | 882 |
