# Supplementary material for: Incidence and risk factors of systemic lupus erythematosus in patients with primary immune thrombocytopenia: a systematic review and meta-analysis
Source: PeerJ. 2024 Apr 22;12:e17152. doi: 10.7717/peerj.17152 (PMC11044880; doi:10.7717/peerj.17152)
Supplement: Supplemental Information 3 [file peerj-12-17152-s003.pdf]

The rationale for conducting the systematic review / meta-analysis

Immune thrombocytopenia (ITP) is the most common bleeding disorder characterized by simple thrombocytopenia. Some studies have demonstrated higher incidences of bleeding, thrombosis, infection, cardiovascular disease, and hematological cancer in ITP patients than in the general population. Systemic lupus erythematosus (SLE) is a chronic autoimmune disease characterized by autoantibody production and multiorgan involvement, which involves multiple systems and organs. Several articles have demonstrated that ITP and SLE share a common genetic predisposition, and some patients with primary ITP may develop SLE during follow-up. However, there are few related studies and the predictive risk factors remain unclear. It is pressingly urgent to clarify the clinical features of those patients who developed SLE from primary ITP. Therefore, we conducted this study to analyzed the incidence and risk factors of SLE in primary ITP patients to provide a scientific basis for clinical treatment and prevention.

The contribution that it makes to knowledge in light of previously published related reports, including other meta-analyses and systematic reviews

In this study, we discovered that 3.02% of patients with primary ITP developed SLE. Additionally, this meta-analysis concluded risk factors for primary ITP development into SLE during follow-up, such as being female, older age, positive ANA, hypocomplementemia, chronic ITP, and bleeding from internal organs. It may be helpful for clinicians to regularly monitor SLE-related indicators in high-risk groups and develop individualized prevention and management interventions.
